# Impact of Starting an Emergency Medicine Residency Program on Overall Mortality Rate in a Regional Trauma Center

**DOI:** 10.14740/jocmr2410w

**Published:** 2015-12-28

**Authors:** Thomas McLaughlin, Osbert Blow, John Herrick, Peter Richman

**Affiliations:** a Department of Emergency Medicine, CHRISTUS Spohn Hospital Corpus Christi - *Memorial*, 2606 Hospital Blvd., Corpus Christi, TX 78405, USA; b Department of Acute Care and Surgical Critical Care, Trauma and Surgical Critical Care Services, CHRISTUS Spohn Hospital Corpus Christi - *Memorial*, 2606 Hospital Blvd., Corpus Christi, TX 78405, USA

**Keywords:** Trauma, Emergency medicine residents, Patient outcomes

## Abstract

**Background:**

CHRISTUS Spohn Hospital Corpus Christi - *Memorial* began an Emergency Medicine Residency Program in March 2007. During each of the three years of their residency, residents are required to complete a trauma surgery rotation. These emergency medicine residents are the only residents participating on this rotation as there is no surgical residency. The Department of Acute Care Surgery, Trauma and Surgical Critical Care analyzed the impact of the residents on trauma patient care outcomes with the hypothesis that there were no differences.

**Methods:**

Data including length of stay in the hospital, length of stay in the intensive care unit, time spent in the emergency department (ED), morbidities and mortalities were compiled from the trauma registry for patients from the year before the residents began (March 1, 2006 to February 28, 2007) and compared with patients from the first year the residents began their trauma rotations (March 1, 2007 to February 29, 2008). T-tests and Mann-Whitney U tests were used to compare continuous variables and a Chi-square test was used to analyze the categorical variable (mortality). Linear and logistic regression analyses were also performed in order to adjust for potential confounding factors.

**Results:**

Trauma patient admission rates were 1,316 before and 1,391 after the residents began. No statistically significant differences were found among all of the outcome variables during the two time periods except for time spent in the ED (P = 0.00), which increased during the year the residents began (236.83 ± 4.53 minutes in 2006 compared to 297.40 ± 5.55 minutes in 2007). Linear and logistic regression analyses confirmed these results with the exception of a statistically significant decrease in mortality with the residents on the trauma service (2.8% in 2006 and 2.1% in 2007, P = 0.00) after adjustment for multiple confounding factors.

**Conclusion:**

The addition of emergency medicine residents to the trauma care service did increase ED length of stay, but did not increase overall hospital or intensive care unit length of stay. There was a statistically significant decrease in adjusted morbidity and mortality, thus supporting our hypothesis that the residency program did not negatively impact the trauma service and its goals of high quality patient care.

## Introduction

Historically, medical residency training programs have been suspected of reducing the level of quality patient care and increasing hospital costs. Medical residents begin their first year of residency lacking significant patient care experience. As a result, residents are suspected of increasing intensive care unit (ICU) and overall hospital length of stay, morbidities and mortalities for patients, and patient costs [1]. Many studies have analyzed the effect of residents by comparing patient outcomes and hospital costs between teaching and non-teaching hospitals. Papanikolaou et al [1] did a systematic review on 132 studies on this topic and found that, overall, teaching hospitals did not improve or worsen patient outcomes (mortality and other patient outcomes) when compared to non-teaching hospitals.

Another concern with residency programs that has been extensively studied is the “July Phenomenon”, wherein each July, senior residents graduate, and a new group of residents enter as interns on various rotations. This drop in overall resident experience level is a difficult adjustment period during which more negative patient outcomes are suspected to occur in teaching hospitals. Twelve studies [2-13], shown in Table 1, have analyzed the July Phenomenon by comparing various patient outcomes early in the medical academic year to later in the year and eight studies found no statistically significant change. Four studies [3, 4, 11, 12] found an increase in patient length of stay, increase in errors for internal medicine patients, increase in near-miss lab request errors and worse shunt surgery outcomes.

**Table 1:** Results of “July Phenomenon” Studies

Year	Author	Outcome
1989	Buchwald et al [2]	No change
1990	Rich et al [3]	Increase in LOS, no other changes
1993	Rich et al [4]	Increase for internal medicine patients, no other changes
1995	Shulkin et al [5]	No change
2001	Claridge et al [6]	No change
2003	Barry et al [7]	No change
2003	Myles [8]	No change
2004	Borenstein et al [9]	No change
2004	Finkielman et al [10]	No change
2005	Chow et al [11]	Increase in near miss errors in lab blood test requests
2006	Kestle et al [12]	Change for some outcomes related to shunt surgery
2007	Ford et al [13]	No change

The purpose of our study is to analyze the specific effect that our new emergency medicine residency program had on trauma patient care outcomes in our hospital. Currently, there are no studies on the impact of emergency medicine residents on trauma patient care, but Offner et al [14] studied the impact of surgical residents on trauma patient care and found that the number of mortalities with and without surgical residents was not statistically significantly different and time spent in the emergency department (ED) and hospital length of stay were statistically significantly shorter during the time period when residents were involved in patient care. There is no general surgery residency located at CHRISTUS Spohn Hospital Corpus Christi providing an opportunity to assess the impact of an emergency medicine residency on the care of trauma patients. The hypothesis for our study was that the addition of the emergency medicine residents would have no effect on the patient outcomes studied.

## Materials and Methods

The Department of Acute Care Surgery, Trauma and Surgical Critical Care at CHRISTUS Spohn Hospital Corpus Christi - *Memorial* is the only designated Level II Trauma Center in the Texas Coastal Bend region and serves a 12-county area. A Family Medicine Residency Program in collaboration with Texas A&M Health Science Center has been present in the hospital since 1973 but the Emergency Medicine Residency Program was a new addition to the Graduate Medical Education Department in March 2007. During their 3-year residency, emergency medicine residents are required to complete a trauma rotation in each of the three years of residency training. The rotation is 4 weeks long during the first and third years and 8 weeks long during the second year and residents work 9 - 12 h shifts within the regulated 80-h work week. The emergency medicine residents are involved in all aspects of trauma patient care throughout their rotations beginning with patient arrival in the ED to transfer to the ICU or trauma floor, and surgical procedures in the operating rooms through patient discharge.

The Department of Acute Care Surgery, Trauma and Surgical Critical Care at CHRISTUS Spohn Hospital Corpus Christi - *Memorial* has maintained a trauma patient registry since 1994 and includes pertinent information regarding a patient’s hospital course from pre-hospital through discharge. The registry includes all patients that meet the trauma registry criteria of having at least one ICD-9 diagnosis code ranging from 800 to 959.9 excluding 905 - 909.9, 910 - 924.9, and 930 - 939.9. Patients included in the registry must have been admitted to the hospital, transferred into the hospital from another hospital or have died from a traumatic injury [15]. Data from the registry are submitted quarterly to the Texas state trauma registry and were used to create a dataset including patient date of admission, age, number of days in the ICU, number of days in the hospital, time spent in the ED, mechanism of injury (blunt, penetrating, thermal), injury severity score (ISS), morbidities and mortality. The ISS extracted from the registry was calculated using the 1990 revision of the abbreviated injury scale. These data sets were compiled for the year before the emergency medicine residents began rotating on the trauma service, March 1, 2006 to February 28, 2007 (2006) and for the first year the residents began rotations on the trauma service, March 1, 2007 to February 29, 2008 (2007).

Patient outcomes studied for this analysis included hospital length of stay, ICU length of stay, morbidities, mortality, and time spent in the ED. These five variables were analyzed before and after the residents began their trauma rotations using *t*-tests for normally distributed data and Mann-Whitney U tests for non-parametric data for continuous variables (hospital length of stay, ICU length of stay, morbidities and time in the ED) and a Chi-square test for the categorical variable (mortality). Linear regression and logistical regression were also performed for the five outcome variables in order to adjust for confounding factors: age of the patient, injury severity score, and mechanism of injury.

Additional analyses were performed by analyzing the five outcome variables stratified by ISS. The three categories were: ISS of 1 - 14, 15 - 24, and 25 and higher. T-tests, Mann-Whitney U tests, and Chi-square tests were used to analyze data for each ISS category. The morbidity variable includes the top eight morbidities found in our registry: pneumonia, a delay in treatment, a return to the ICU, deep vein thrombosis, reintubation, decubitus ulcers, urinary tract infection, and wound infection. The variable excludes all other morbidities that were not frequently found in our registry. All statistical analyses were performed using SPSS for Windows Version 16.0. A value of P < 0.05 was determined to be statistically significant. This study was accepted by the CHRISTUS Spohn Institutional Board of Review in August 2008.

## Results

During 2006, 1,316 hospital admissions were entered into the trauma registry at CHRISTUS Spohn Hospital Corpus Christi - *Memorial* and 1,391 patients were entered during 2007. Patients who had ED arrival and/or departure time information missing were excluded from the time spent in ED analyses, so that n = 1,110 for 2006 and n = 1,293 for 2007 for the analyses for that variable. Mean ± SEM and percentages of demographic characteristics of patients for 2006 and 2007 are shown in Table 2. Average patient age and mechanism of injury were similar between 2006 and 2007: average age in 2006 was 40.36 ± 0.50, average age in 2007 was 40.21 ± 0.48 (P = 0.97), 82.3% of patients had blunt injuries in 2006, and 80.4% of patients had blunt injuries in 2007 (P = 0.78). Average ISS was statistically significantly higher during the first year residents were on the trauma service compared to the year before residents arrived: 8.27 ± 0.23 in 2006, 9.05 ± 0.23 in 2007 (P = 0.02). However, stratification by ISS showed no differences during the two time periods (P = 0.35).

**Table 2:** Demographic Information

	Before residents (n = 1,316)	During residents (n = 1,391)	P value
Age (years)	40.36 ± 0.50	40.21 ± 0.48	0.97
Mechanism of injury			
Blunt (%)	82.3	80.4	
Penetrating (%)	16.2	17.7	0.19
Thermal (%)	1.5	1.9	
Injury severity score (overall)	8.27 ± 0.23	9.05 ± 0.23	0.02
< 15 (%)	82.8	80.7	
15 - 24 (%)	11.3	12.7	0.35
≥ 25 (%)	5.9	6.6	

The outcome variables length of stay in the hospital, length of stay in the ICU, morbidities and mortalities were not statistically significantly different in 2006 and 2007. The amount of time a patient spent in the ED increased when emergency medicine residents were on the trauma service in 2007 and was statistically significantly different from 2006: 236.83 ± 4.53 min in 2006 and 297.40 ± 5.55 min in 2007 (P = 0.00). Table 3 shows averages and P values from *t*-tests, Mann-Whitney U tests and Chi-square tests for similarity of means for the four outcome variables.

**Table 3:** Tests for Similarity of Means for Selected Outcome Variables

	Before EM residents (n = 1,316)	During EM residents (n = 1,391)	P value
Length of stay in ICU (n)	1.88 ± 0.13	2.09 ± 0.14	0.26
Length of stay in hospital (n)	5.28 ± 0.20	5.20 ± 0.20	0.12
Morbidities (%)	7.83	7.26	1.00
Mortalities (%)	2.81	2.01	0.18
Time spent in ED* (min)	236.83 ± 4.53	297.40 ± 5.55	0.00

*Patients that did not have times recorded for ED arrival and departure in their records were excluded for this analysis: before residents (n = 1,110); after residents (n = 1,293).

Multiple regression analyses confirmed these findings with the exception of mortality which was statistically significantly different between the two time periods when adjusted for multiple covariates including age, mechanism of injury and ISS (2.81% in 2006 and 2.01% in 2007, P = 0.01). Length of stay in the hospital, length of stay in the ICU, morbidities and time spent in the ED were consistent with the *t*-tests and Mann-Whitney U tests and the results are found in Table 4.

**Table 4:** Multiple Regression Analysis

	Age-adjusted coefficient	P value	Multiple covariate adjusted coefficient*	P value
Hospital length of stay	-0.01	0.79	-0.02	0.18
ICU length of stay	0.02	0.26	-0.00	0.90
Morbidity	-0.01	0.69	-0.03	0.17
Mortality **	-0.34	0.18	-0.81	0.01
Time in ED	0.17	0.00	0.18	0.00

*Multiple covariate adjusted coefficient and P-value includes age, mechanism of injury and ISS. **Logistic regression analysis was calculated for mortality whereas linear regression analyses were calculated for the hospital length of stay, ICU length of stay, morbidity, and time in ED.

ISS stratification into three groups was performed to determine the presence of effect modification. ISS categories were determined *a priori* based on ISS cut points used in previous studies. Overall, no statistically significant differences were established in ICU length of stay, morbidity and mortality when stratified by ISS category. A statistically significant difference was noted in hospital length of stay for ISS category 1 - 14 (P = 0.01), but there was no difference in more severely injured patients with ISS 15 and higher. Differences were found in the lower ISS categories for time spent in the ED. For both categories ISS < 15 and ISS 15 - 24, the time spent in the ED increased in 2007 and was statistically significantly different (P = 0.00 for both), but the category ISS ≥ 25 was not (P = 0.18). This shows evidence of effect modification by ISS for length of stay in the hospital and time spent in ED. Table 5 shows the complete results of the ISS stratification.

**Table 5:** Similarity of Means Stratified by Injury Severity Score

	N (before, during residents)	Before EM residents	During EM residents	P value
Length of stay in ICU (n)				
ISS < 15	1,090; 1,122	1.03 ± 0.09	1.09 ± 0.09	0.06
ISS 15 - 24	148; 177	4.57 ± 0.62	4.59 ± 0.45	0.88
ISS ≥ 25	77; 92	8.60 ± 1.24	9.48 ± 1.24	0.61
Length of stay in hospital (n)				
ISS < 15	1,090; 1,122	4.27 ± 0.15	4.13 ± 0.17	0.01
ISS 15 - 24	148; 177	8.62 ± 0.88	8.16 ± 0.62	0.91
ISS ≥ 25	77; 92	12.91 ± 1.64	12.57 ± 1.43	0.81
Morbidities (%)				
ISS < 15	1,090; 1,122	3.0%	2.0%	0.38
ISS 15 - 24	148; 177	20.0%	19.0%	0.99
ISS ≥ 25	77; 92	53.0%	51.0%	0.90
Mortalities (%)				
ISS < 15	1,090; 1,122	0.3%	0.0%	0.30
ISS 15 - 24	148; 177	5.0%	3.0%	0.54
ISS ≥ 25	77; 92	35%	23.0%	0.08
Time spent in ED (min)				
ISS < 15	918; 1,040	240.99 ± 4.89	312.06 ± 6.39	0.00
ISS 15 - 24	130; 168	227.45 ± 15.72	275.27 ± 13.03	0.00
ISS ≥ 25	66; 89	182.80 ± 15.22	154.44 ± 10.46	0.18

## Discussion

The results of our analyses show that length of stay in the hospital, length of stay in the ICU, and morbidities were not affected by the addition of the emergency medicine residents to the trauma service. The decrease in mortality in 2007 was statistically significant after adjustment for multiple covariates. Evidence of effect modification by time spent in the ED was shown by a statistically significant increase in ISS groups 1 - 14 and 15 - 24. Hospital length of stay was also statistically significantly decreased in ISS category 1 - 14 which is also evidence of effect modification.

While there are no directly similar studies to compare our results to, our findings are similar to the majority of studies that have analyzed the July Phenomenon or compared teaching and non-teaching hospitals [1-13] in that most of the variables studied showed no difference with the addition of the residency program. In comparison to Offner et al [14] and their study on surgical residents’ impact on the trauma service, our study has somewhat different results as they found a statistically significant decrease in length of stay in the hospital, length of stay in the ICU, and time in the ED whereas only our test for time in the ED statistically significantly increased. Our non-statistically significant decrease in mortality with the addition of residents was similar to their results but our linear regression test showed a significant result after the adjustment for multiple covariates.

Emergency medicine residents and surgical residents have different responsibilities in trauma patient care. Surgical residents spend 4 years assisting in the treatment of patients admitted to the trauma service whereas emergency medicine residents spend only a total of 4 months over the 3 years of their residency. While there are emergency medicine residents involved in the trauma service on a rotated schedule consistently, they do not have the constant long-term experience with trauma patients that the surgery residents have. This does not allow them to gain as much experience as the surgical residents and could explain the difference in the results of our study compared to Offner et al [14].

The decrease in mortality of trauma patients in 2007 could be due to the fact that the presence of the emergency medicine residents on the trauma service in rounds and at patient bedsides stimulated additional discussion in care plans. The increase in time spent in the ED with the presence of the residents on the trauma service could be a result of the emergency medicine residents’ thoroughness in patient evaluation and perhaps some lack of confidence in skills set. The lack of a significant statistical difference in patients with ISS ≥ 25 is supported by the fact that these patients entered our facility as a trauma code. According to ACSCOT guidelines, the surgical attending must be present at the time of patient arrival for trauma codes which is the protocol at CHRISTUS Spohn Hospital Corpus Christi - *Memorial*. However, emergency medicine residents have more interaction with trauma patients with ISS < 25 as the residents are the first providers for less critically injured trauma patients in the ED.

Limitations of the study include the inability to adjust for factors involved in patient care that are not collected in the trauma registry such as nursing and physician staff changes, equipment upgrades used for treatment, and variation in specific diagnoses. Trauma patients are admitted with a broad spectrum of injuries that are difficult to classify into similar groups. Patients with the same diagnosis may have different courses of treatment and outcomes depending on the specific mechanism, location and severity of the injury. Adjustment by ISS helps with this but it cannot fully allow for direct comparisons.

There were several changes in nursing and physician staff and equipment during the 2 years of this study. In 2006, there were nursing staff shortages on the hospital floor units. The hospital also underwent renovations in the units which caused one half of a unit to be closed at a time. This caused a shorter supply of beds during 2006. In September 2006, CHRISTUS Spohn Hospital Corpus Christi - *Memorial* hired a new Medical Director of Trauma and Surgical Critical Care Services but no other surgeon staff changes were made during the 2-year period. The hospital burn unit was closed at the end of 2006 and subsequently all burn patients were transferred out of the facility. All equipment and technology used in the care of patients during the 2 years remained the same except for the addition of a DH4 Helmer Quick Thaw device in August 2007.

Strengths of the study include the large number of patients during the two time periods analyzed and good reliability of data from the trauma registry. The registry is complete and accurate information on the patients who meet the trauma registry criteria as the registry received zero rejected records from the state registry submission for the past year. The two trauma registrars have taken the Trauma Register Course Basic and Advanced taught by the American Trauma Society which focused on overall registry data input and ICD-9 concepts. The registrars also completed the Injury Scaling; Uses and Techniques course taught by the Association for the Advancement of Automotive Medicine which teaches ISS scoring. An additional strength of the study is that nursing turnover rates for the ICU and the ED were less than 10% and zero respectively for the years 2006 - 2007.

In conclusion, we found that patient length of stay in the hospital, patient length of stay in the ICU, and patient morbidity were not affected by the addition of the emergency medicine residents to the trauma service. Mortality statistically significantly decreased after adjustment for covariates. In addition, the time spent in the ED statistically significantly increased in ISS groups < 15 and 15 - 24 in 2007 compared to 2006. Therefore, the presence of emergency medicine residents did not negatively impact trauma patient care and may be responsible for the decrease in overall mortality. Future studies on this topic should analyze the impact of emergency medicine residents on other hospital trauma services that have similar or higher trauma level designations. Other studies should focus on the impact of emergency medicine residents on other hospital departments.
